# PuraMatrix Encapsulation of Cancer Cells

**DOI:** 10.3791/1692

**Published:** 2009-12-17

**Authors:** Adnan O. Abu-Yousif, Imran Rizvi, Conor L. Evans, Jonathan P. Celli, Tayyaba Hasan

**Affiliations:** Wellman Center for Photomedicine Massachusetts General Hospital, Harvard Medical School; Thayer School of Engineering, Dartmouth College; Department of Dermatology, Harvard Medical School

## Abstract

Increasing evidence suggests that culturing cancer cells in three dimensions more accurately recapitulates the complexity of tumor biology. Many of these models utilize reconstituted basement membrane derived from animals which contain a variable amount of growth factors and cytokines that can influence the growth of these cell culture models. Here, we describe in detail the preparation and use of PuraMatrix, a commercially available self assembling peptide gel that is devoid of animal-derived material and pathogens to encapsulate and propagate the ovarian cancer cell line, OVCAR-5. We begin by describing how to prepare the PuraMatrix prior to use. Next, we demonstrate how to properly mix the PuraMatrix and cell suspension to encapsulate the cells in the hydrogel. Upon the addition of cell culture media or injection into a physiological environment, the peptide component of PuraMatrix rapidly self assembles into a 3D hydrogel that exhibits a nanometer scale fibrous structure with an average pore size of 5-200 nm^1^. In addition, we demonstrate how to propagate cultures grown in encapsulated PuraMatrix. When encapsulated in PuraMatrix, OVCAR-5 cells assemble into three dimensional acinar structures that more closely resemble the morphology of micrometastatic nodules observed in the clinic than monolayer in vitro models. Using confocal microscopy we illustrate the appearance of representative OVCAR-5 cells encapsulated in PuraMatrix on day 1, 3, 5, and 7 post plating. The use of PuraMatrix to culture cancer cells should improve our understanding of the disease and allow us to assess treatment response in more clinically predictive model systems.

**Figure:**
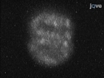


## Protocol

### Technical Notes:

- Gelation is initiated by the addition of salts. Therefore, it is CRITICAL that no salts come in contact with PuraMatrix until gelation is desired; exposure to salts of very low ionic strength (e.g., < 0.05 M) will cause irreversible gelation.
- Leftover stock solutions that are prepared can be used at a later date provided they have not come in contact with salts. You do not need to worry about repeat sonication.
- Air bubbles can be removed aliquoting PuraMatrix into sterile tubes followed by centrifugation.
- Be very careful during media changes as aspiration can easily disrupt the PuraMatrix bed.

### Materials:

BD PuraMatrix RAD16 Peptide Hydrogel-1% (Cat # 354250): The total peptide concentration of the undiluted mixture is 10 mg/ml.

- Water Bath Sonicator or Vortex
- 96-well cell culture dish
- Cell Culture Media
- Sterile Filtered H_2_O
- Sterile Filtered 20% Sucrose
- Cells (all at 5,000 cells/well): OVCAR-5

All steps must be performed under aseptic conditions.

### Procedure:

1. Sonicate the 1% PuraMatrix (RAD16) in a water bath sonicator for 30 minutes to reduce the viscosity prior to use.
2. The 1% PuraMatrix solution can now be aliquoted into sterile tubes to simplify future experiments.
  1. Avoid the formation of air bubbles; if they form simply spin down the sterile tubes containing PuraMatrix at 1000 rpm (300 x g) for 5 minutes.
  2. The total peptide concentration of the undiluted mixture is 10 mg/ml.
3. Two sets of 4 wells will be plated at 2.5 mg/ml and 1.25 mg/ml total peptide concentration, respectively.
4. Dilute the PuraMatrix in each well set with 0.2 μm sterile filtered 20% sucrose and 13.3% sucrose to total peptide concentrations of 5 mg/ml and 2.5 mg/ml, respectively to achieve a 2x concentration of BD PuraMatrix in 10% sucrose.
  1. To Make 20% sucrose, dilute 10 g of sucrose, then volume to 50 ml water.
  2. 13% and 10% sucrose can be prepared by diluting the 20% stock solution.
    1. 13% Sucrose (for every 10 ml):  6.5 ml 20% sucrose + 3.5 ml sterile H_2_O.
    2. 10% Sucrose (for every 10 ml): 5.0 ml 20% sucrose + 5.0 ml sterile H_2_O.
5. Prepare a master mix of PuraMatrix peptide based on the number of wells you intend to plate. (See example below.) Then aliquot into individual tubes containing 25 ml of the master mix.
  1. The master mix should be prepared at twice the desired final concentration of total peptide as it will be diluted with an equal volume of cells during plating.
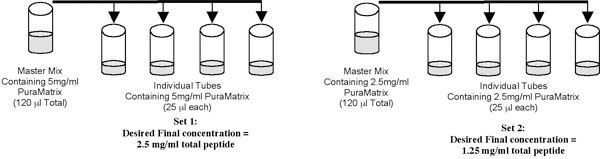
6. Prepare cell suspensions by trypsinizing cell monolayer cell cultures.
  1. 2 ml of 0.05% Trypsin EDTA can be used for each T-75 Flask.
7. Neutralize the trypsin by addition of cell culture media containing 10% heat inactivated fetal bovine serum (use at least 2 ml of media per ml of trypsin used in the previous step.)
8. Centrifuge the cells at 1000 rpm (~ 300 x g) for 5 minutes to create a cell pellet.
  1. Resuspend the cells in 10% sterile filtered sucrose.
  2. Count the cells and spin down again at 1000 rpm (~ 300 x g) for 5 minutes to remove trace amounts of salt found in the media.
9. Resuspend the OVCAR-5 cells in 10% sucrose at 2.0 x 10^5^ cells/ml so that the final cell count per well will be 5,000 cells in each well. **The following steps should be performed rapidly.**
10. Add 25 μl of the 2.0 x 10^5^ cells/ml suspension to the first individual tube containing 25 μl of the master mix of PuraMatrix as shown in the above schematic.
  1. Quickly mix by pipetting (~5 times up and down) in the tube without introducing air bubbles.
  2. Then pipette the 50 μl cell suspension/PuraMatrix mixture into the appropriate well of a 96 well dish.
  3. Initiate gelation of the BD PuraMatrix by gently pipetting 150 μl cell culture media (RPMI + 10% FBS)  to the side of the well
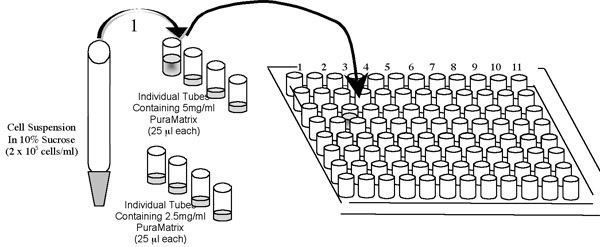
11. Repeat the steps outlined in 8 until all wells have been plated.
12. After 30 minutes **Very Gently** remove ~2/3 of the media using a 200 μl pipette to equilibrate the pH of the hydrogel.
  1. **DO NOT USE A VACUUM ASPIRATOR AS THIS WILL DISRUPT THE MATRIX**
  2. **Place the pipette tip in the well so that it does not touch the PuraMatrix bed and gently remove media from the well.**
  3. **Care should be taken to not disrupt the matrix during the culture of cells in PuraMatrix.**
13. **Very Gently** add 150 μl of fresh cell culture media to the side of the well containing cells encapsulated in PuraMatrix.
14. Change the cell culture media in each well every 48 hours with fresh culture media by carefully repeating steps 10 and 11.

### Representative Results:


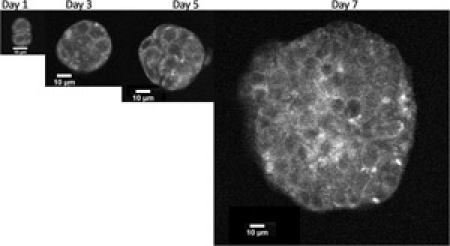
 **Figure 1.** OVCAR-5  cells were encapsulated as described in the above protocol.  Images were acquired using an inverted Olympus FV1000 microscope equipped with Spectra-Physics DeepSee Ti:Sapphire laser tuned to 750 nm.  Imaging sessions were conducted on days 1, 3, 5 and 7 post-plating.  A long working distance, water dipping 40x objective (Olympus LUMPLFLN 0.8 NA) was used to image through the PuraMatrix.  Three-dimensional volumes were collected by acquiring image stacks in 1.47 μm axial steps. Two non-descanned detectors collected the autofluorescence emission using violet (440-490 nm) and green (510-550 nm) bandpass filters.  The final images and depth stack movies were processed using ImageJ by combining both detection channels. Please click here to see a larger version of figure 1.
